# Adapting the American Community Survey for the Affordable Care Act

**DOI:** 10.1111/1475-6773.70066

**Published:** 2025-11-04

**Authors:** Joanne Pascale, Angela R. Fertig

**Affiliations:** ^1^ U.S. Census Bureau Washington District of Columbia USA; ^2^ Humphrey School of Public Affairs, University of Minnesota Minneapolis Minnesota USA

**Keywords:** ACS, Affordable Care Act, health insurance, marketplace, reporting accuracy

## Abstract

**Objective:**

To measure the accuracy of questions on health insurance premiums and subsidies added to the American Community Survey (ACS) and their utility in categorizing coverage type following the Affordable Care Act (ACA).

**Study Setting and Design:**

A reverse record check study where households in Minnesota with individuals enrolled in five different types of coverage—employer‐sponsored insurance (ESI), non‐group (outside the marketplace), marketplace, Medicaid and MinnesotaCare (a public plan requiring premium contributions from the enrollee)—were administered a telephone survey that included the ACS health insurance module appended with experimental questions on premiums and subsidies.

**Data Sources and Analytic Sample:**

Enrollment records from a private insurer were used as the sample for primary survey data collection in the spring of 2015 using the ACS health insurance module. Survey data were matched back to enrollment records, which indicated coverage status at the time of the survey. The analytic sample includes matched data on about 600 individuals.

**Principal Findings:**

In total, 100%, 95.3%, and 86.9% of marketplace, non‐group, and ESI enrollees, respectively, were correctly reported to have a premium. 74.6% of Medicaid enrollees were correctly reported NOT to have a premium and 77.4% of MinnesotaCare enrollees were correctly reported to HAVE a premium. For the subsidy item, correct reports of no subsidy were 99.1%, 93.8%, and 80.9% for ESI, non‐group, and unsubsidized marketplace enrollees, respectively. A total of 72.4% of subsidized marketplace enrollees were correctly reported to have a subsidy. Analysis also indicates that an algorithm leveraging these two new data points can be used to separate the overall “direct purchase” category into two sub‐groups: subsidized marketplace and unsubsidized marketplace combined with individual non‐group.

**Conclusions:**

Results indicate high levels of reporting accuracy for questions about premiums and subsidies. Thus, this post‐ACA module of the ACS is capable of rendering more detailed coverage types than previously possible.

**Summary**

- What is known on this topic
  ○ The American Community Survey introduced questions about health insurance premiums and subsidies in 2019 to better capture health insurance coverage type post‐Affordable Care Act.
  ○ Responses to health insurance survey questions vary in accuracy due factors such as question wording, context within the questionnaire, and the complexity of the health insurance landscape in the U.S.
- What this study adds
  ○ Reporting accuracy on these new items is high; 86+% with premiums were accurately reported to have a premium, and 72% of subsidized marketplace enrollees were reported to have a subsidy.
  ○ The two items can allow researchers to categorize individuals' health insurance coverage with greater granularity, for example, subsidized marketplace coverage can be distinguished from non‐group coverage without subsidies.

## 1 Introduction

Nearly 100 million Americans received subsidized health insurance coverage in 2025 through government programs (e.g., Medicaid) or private coverage (e.g., Marketplace with subsidies via tax credits) [1]. Because both Medicaid and Marketplace health insurance coverage are joint federal‐state programs, their funding and rules regarding eligibility, enrollment procedures and administrative policies are subject to change based on the shifting political landscape. For example, the One Big Beautiful Bill Act passed in July 2025 and the federal government shutdown of October 2025 both represent struggles over subsidies for health insurance through marketplace plans enacted by the 2010 Affordable Care Act (ACA) [2] and cuts to Medicaid for certain groups [3]. It is critical to have a data source that can identify whether people have Medicaid or Marketplace coverage, and if the latter, whether the premiums are subsidized, to enable analysis of the relationship between coverage status and various outcomes. While there are administrative records on Medicaid and Marketplace enrollments, these data sources are generally lacking in characteristics of enrollees such as employment, health, family composition, and income status, that are needed to understand the affected populations. The American Community Survey (ACS) includes a rich array of characteristics and a sample size large enough to examine variations within and across states [4].

For these reasons, the ACS has included a health insurance module since 2008 [5, 6, 7]. Two years later the ACA was passed, which called for introducing new marketplace coverage in 2014. To prepare for that, research on adapting the ACS for the ACA began in 2011 with a study in Massachusetts, which had a state‐level marketplace on which the national ACA was partially modeled. Quantitative and qualitative results from that early testing suggested that the ACS module could be maintained intact, and additional questions on features of coverage—including premiums and subsidies—could be appended to the end of the module in order to measure and categorize coverage type post‐ACA [8]. After additional testing [9], questions on premiums and subsidies were added to the ACS in 2019 [10]. In this study we examine the reporting accuracy of the new data points using data from a validation study of the ACS, and we explore how to best leverage these data points to sharpen coverage type categorization in the ACS.

## 2 Methods

### 2.1 Study Design

The data for this study come from an experimental study called CHIME (Comparing Health Insurance Measurement Error). CHIME is a reverse record check study in which enrollment records from a large private health insurer in Minnesota were used as the sample source for a telephone survey that included the ACS health insurance module. Specifically, the health insurer provided randomly selected samples of enrollees in three types of private coverage (employer‐sponsored insurance (ESI) and non‐group both on and off the marketplace) and two types of public coverage (Medicaid and MinnesotaCare, which is Minnesota's Basic Health Plan with a sliding scale premium). Policyholders were eligible to be sampled if they were under age 65 and enrolled when the sample was drawn in December 2014. Phone numbers associated with these households were administered a short (17‐min) telephone survey in the spring of 2015 by Census callers. In order to set the context for the ACS health insurance module, and also to collect data for covariate analysis, the survey included questions on typical demographics (e.g., age, sex, and race), labor force participation, and unearned income (e.g., social security, SNAP (aka Food Stamps), unemployment compensation).

Survey data and the enrollment records were then linked in order to evaluate the concordance between the two. The enrollment records were extracted after the surveys were complete and included coverage type at the time of the survey (ESI, subsidized marketplace, unsubsidized marketplace, Medicaid, or MinnesotaCare), whether a premium was paid, and, for Marketplace plans, whether the premium was subsidized, along with matching variables (e.g., phone number, address, name, and birthdate). For convenience we refer to the concordance between the survey reports and the enrollment records as “reporting accuracy” though we acknowledge that there could be errors in the enrollment records and/or in the linking process. Extensive data cleaning and processing conducted by informatics staff handling the enrollment records likely minimized these types of errors [11]. The final analysis file included about 1800 person records and data were weighted to reflect the population of the health insurance provider within each stratum. More details on the CHIME study methodology can be found elsewhere [11, 12, 13].

### 2.2 Questionnaire

The ACS module includes eight questions, each on a different type of coverage, as shown in Table 1. In the CHIME questionnaire, we mimicked those eight questions and added the premium and subsidy questions (Questions 10 and 11, respectively) and also a question on the portal/marketplace (Question 9). However, in this analysis, we focus only on the premium and subsidy items because these are the only two questions that were ultimately added to the ACS module in 2019.

**TABLE 1:** CHIME health insurance questionnaire module.

(1) Are you currently covered by health insurance through a current or former employer or union of yours or another family member?
(2) Are you currently covered by health insurance purchased directly from an insurance company by you or another family member?
(3) Are you currently covered by Medicare, for people age 65 or older or people with certain disabilities?
(4) Are you currently covered by Medicaid, Medical Assistance, or any kind of government‐assistance plan for those with low incomes or a disability?
(5) Are you currently covered by TRICARE or other military health care?
(6) Are you currently covered through the Veteran's Administration or have you ever used or enrolled for VA health care?
(7) Are you currently covered through the Indian Health Service?
(8) Are you currently covered by any other health insurance or health coverage plan? [If yes, open‐text specify]
(9) Was this plan obtained through a State or Federal Marketplace, Healthcare.gov, or a similar state website?
(10) Do you or another family member pay a premium for this health insurance plan? A premium is a fixed amount of money paid on a regular basis for health coverage. It does not include copays, deductibles, or other expenses such as prescription costs.
(11) Based on family income, do you or another family member receive financial assistance through a subsidy or tax credit to help pay part or all of the cost of the premium for this plan?

*Note:* Response options for all questions are Yes, No, Don't Know (D), and Refused (R).

The ACS series is asked at the “person level,” meaning the entire series (Questions 1–8) is administered for the first person listed on the household roster, and then the series is repeated for the second person on the roster, and so on, until all household members have been asked about. We wanted to mimic the ACS module as it was in 2015, but we also wanted to examine the reporting accuracy of the experimental questions on premiums and subsidies. To do that, and also avoid “contaminating” the series by adding these experimental questions in the middle of the series that could affect later responses, we added the experimental items to the series for only the last person on the household roster. Furthermore, these items were only asked if at least one type of coverage was reported for that last person on the household roster. Thus, Questions 1–8 were asked about all household members, and Questions 9–11 were only asked of the last person listed on the household roster, if coverage was reported for that person.

There are some differences between the sample that was asked the premium and subsidy questions (hereafter called the “PS” sample) (about 600 individuals) and the sample that was not (about 1200 individuals). To explore whether these samples differed in important ways, we compared the demographic profile of each (complete results shown in Supporting Information: Appendix A). As expected, given that the PS items were asked about the last person in the household, and because respondents tend to list household members by oldest to youngest, the PS sample includes significantly more children compared to the individuals who did not receive the PS items. The PS sample also includes significantly more single‐person households, which by definition are self‐reports (versus proxies). Both of these characteristics are associated with higher reporting accuracy [14, 15].

### 2.3 Analytic Approach

The main metric in the analysis is “sensitivity,” where we begin with a measure of characteristic X from the enrollment records and compare that to the survey report of that same characteristic. Specifically, if the enrollment records indicate there is a premium associated with the coverage, how often is a premium reported in the survey? We do the same for the subsidy item. For the premium item, all ESI, non‐group, marketplace and MinnesotaCare plans carried a premium, so the correct survey answer for enrollees in these plans is “yes.” For Medicaid, the program in Minnesota at the time of data collection did not carry a premium, and so the correct survey answer is “no.” For the subsidy item, all ESI, non‐group and unsubsidized marketplace enrollees were unsubsidized, so the correct survey answer is “no.” For the subsidized marketplace enrollees, the correct answer is “yes.” For public coverage the correct answer to the subsidy item is debatable. For Medicaid there is no premium; hence a question on whether the premium is subsidized is irrelevant (however if a respondent indicated that a premium was paid, the subsidy question was asked). For MinnesotaCare, which is a subsidized health care plan for low‐income households and requires a premium, the term “subsidy” is not explicit for enrollees. Thus, for public coverage of either type, there is not a clear right answer for the subsidy item.

**FIGURE 1:**
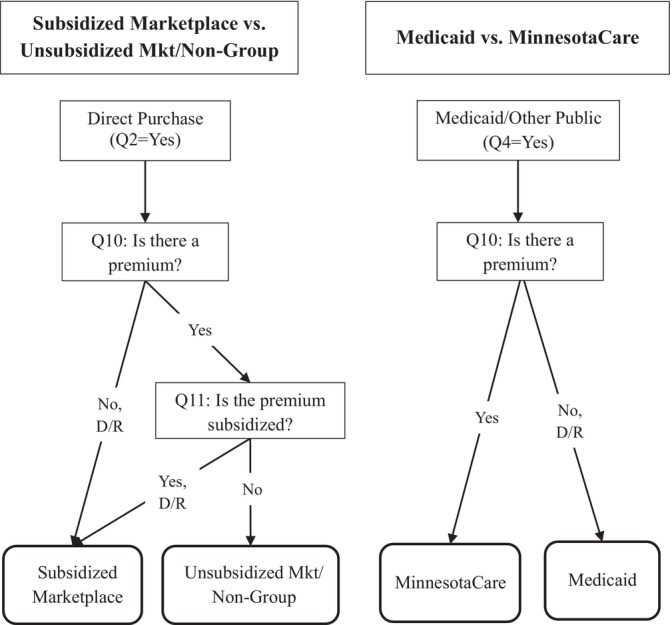
Using premium and subsidy to categorize coverage type in the ACS. *Note:* D/R, Don't know/Refused; Mkt, marketplace; Q, Question.

Next, we examined the accuracy of combining the premium and subsidy items with the core ACS module (Questions 1–8 above) to produce more detailed categories of coverage type than possible without these items. Figure 1 displays the logic used to combine items. Regarding private coverage, we begin with the universe of respondents who were reported to have directly purchased coverage (Question 2 above). Among that pool, those who were reported to have no premium or a subsidized premium were categorized as subsidized marketplace enrollees, while those reported to have an unsubsidized premium were categorized as either unsubsidized marketplace or non‐group (outside the marketplace). These two types of coverage could not be separated because we do not have an indicator of whether the plan was obtained on the marketplace (e.g., healthcare.gov) or whether it was directly purchased outside the marketplace. Regarding public coverage, we begin with the universe who were reported to have Medicaid or another government assistance plan (Question 4 above). Among that pool, those who were reported to have a premium were categorized as MinnesotaCare enrollees, while those reported to have no premium were categorized as Medicaid enrollees. Finally, we compare the weighted prevalence of coverage types in the records to estimates of self‐reports.

Regarding item‐missing data (D/R), levels were very low and given the relatively small sample size we did not systematically impute or weight for non‐response. Thus, we had to make some assumptions. For the premium item we assumed “don't know” and “refused” responses were more likely to actually have no premium, and for the subsidy item we assumed “don't know” and “refused” responses were more likely to actually be subsidized. See Supporting Information: Appendix B for an explanation of how the other/specify write‐ins were handled.

## 3 Results

### 3.1 Reporting Accuracy of Premium and Subsidy Questions

Reporting accuracy for the premium and subsidy survey items is shown in Table 2. For marketplace enrollees (both subsidized and unsubsidized) all were correctly reported to have a premium, and for non‐group enrollees the level was also high at 95.3%. Interestingly, for ESI enrollees the level was lower, at 86.9%. It may be the case that an employer pays the entire premium, making this response accurate for some individuals. Also note that the marketplace samples were smaller than the other strata resulting in greater sampling error, which may explain the perfect accuracy finding. Among public enrollees, for MinnesotaCare 77.4% were correctly reported to have a premium and for Medicaid 74.6% were correctly reported not to have a premium. Item‐missing data was extremely low; zero in the non‐group, marketplace and MinnesotaCare strata and only 0.9% in the Medicaid and 1.5% in the ESI strata.

**TABLE 2:** Reporting accuracy of premium and subsidy items by strata (Weighted).

ACS self‐reports		Coverage type according to enrollment records					
		ESI	Non‐group	Market unsubs	Market subs	Medicaid	MinnesotaCare
Premium	Yes	**86.9**	**95.3**	**100**	**100**	24.4	**77.4**
	No	11.6	4.7	0.0	0.0	**74.6**	22.6
	D/R	1.5	0.0	0.0	0.0	0.9	0.0
	Total	100	100	100	100	100	100
Subsidy	Yes	0.9	6.2	19.1	**72.4**	21.2	21.5
	No	**99.1**	**93.8**	**80.9**	25.7	77.0	69.5
	D/R	0.0	0.0	0.0	1.9	1.7	9.0
	Total	100	100	100	100	100	100

*Note:* Figures in bold large font indicate correct reporting; other font indicates incorrect reporting or missing data (D/*R* = Don't Know/Refused). Due to disclosure avoidance, we do not provide specific sample sizes by strata; see Fertig et al. [11] for more details. Example interpretation: 86.9% of ESI enrollees were reported to pay a premium and 99.1% were reported to not pay a subsidy.

Abbreviations: ACS, American Community Survey; ESI, Employer Sponsored Insurance; Subs, subsidized.

For the subsidy question, reporting accuracy was highest for ESI, with 99.1% correctly reporting there was no subsidy. For non‐group the level was also high, with 93.8% reporting no subsidy. Unsubsidized marketplace enrollees were correctly reported NOT to have a subsidy 80.9% of the time and 72.4% of subsidized marketplace enrollees were correctly reported to HAVE a subsidy. For public coverage we include results in Table 2 but highlight that the meaning of this particular data point is not clear for reasons discussed above. For Medicaid, while there is no premium, 24.4% of Medicaid enrollees misreport that they do pay a premium and thus were asked the subsidy question. For both Medicaid and MinnesotaCare, the majority (77.0% and 69.5%, respectively) reported that their premium was NOT subsidized. We conjecture that public coverage is not marketed or described to enrollees using the term subsidy, unlike marketplace coverage where there is a heavy emphasis on the terms premium and subsidy. Thus, from the perspective of interpreting survey reports, a survey item with the term subsidy is more appropriate for marketplace enrollees than for public plan enrollees. Missing data for this item was very low for most strata (0%–1.9%) but 9.0% for MinnesotaCare.

### 3.2 Reporting Accuracy of Coverage Types Created Using the Premium and Subsidy Items

We now turn to the sensitivity of coverage type when using the premium and subsidy items to categorize type. The rows in Table 3 show results for the basic categories of coverage defined by the plan types asked about in the core health insurance module—specifically ESI, direct‐purchase, Medicare, public, military and other. The rows also show sensitivity for the sub‐categories of coverage that can be created using the premium and subsidy items in tandem with that original module, as shown in the algorithm in Figure 1. The columns in Table 3 represent the universe of enrollees in each stratum. Thus, the cells in the table indicate: for an enrollee of Coverage Type X, how often was Coverage Type X reported for them? And, was another coverage type reported instead of (or in addition to) Coverage Type X? Note that the “Direct/Mkt Unsubsidized” column combines two original strata from the enrollment records: the non‐group and unsubsidized marketplace strata. This is done to match the disaggregated self‐reported category of Direct/Unsubsidized Marketplace. Without a question about whether the health plan was purchased on healthcare.gov or a state‐specific equivalent (like Question 9, which was not added to the ACS in 2019), there is simply no way to distinguish self‐reported non‐group/outside the marketplace from unsubsidized marketplace coverage. So, because these two types of coverage have to be combined on the self‐reported side, we combine them on the enrollment records side as well. Note there is no row for self‐reported uninsured because the PS sample was defined by having reported at least one type of coverage in the core health insurance module.

**TABLE 3:** Reporting accuracy of coverage type, by strata (Weighted).

ACS self‐reports	Coverage type according to enrollment records				
	ESI	Direct/Mkt unsubsidized	Market subsidized	Medicaid	MinnesotaCare
ESI	**96.9**	10.6	1.9	10.2	10.8
Direct	6.1	**90.2**	**92.3**	11.0	47.8
Dir/Mkt Unsubs	*6.1*	***82.1***	*21.9*	*6.7*	*25.1*
Mkt Subs	*0.0*	*8.1*	***70.5***	*4.3*	*22.7*
Public	3.2	4.5	7.7	**83.0**	**54.3**
Medicaid	*0.1*	*2.0*	*0.0*	***69.2***	*17.2*
MinnesotaCare	*3.2*	*2.5*	*7.7*	*13.8*	***37.1***
Medicare	0.0	2.6	1.9	4.0	4.7
Military	0.7	0.9	1.9	0.0	0.0
Other	0.7	2.3	8.4	5.7	7.4
TOTAL	107.6	111.1	114.1	113.9	125.0

*Note:* Figures in bold large font indicate correct reporting. Figures in italics indicate sub‐categories of coverage created using the premium and subsidy items. Totals are greater than 100 because individuals can report multiple coverage types. Due to disclosure avoidance, we do not provide specific sample sizes by strata; see Fertig et al. [11] for more details. Example interpretation: 96.9% of ESI enrollees were accurately reported to have ESI coverage. 6.1% of ESI enrollees were inaccurately reported to have Direct/non‐group coverage.

Abbreviations: ACS, American Community Survey; Dir, direct purchase; ESI, Employer Sponsored Insurance; Mkt, marketplace; Unsubs, unsubsidized.

Results show that for private coverage sensitivity is above 90%. As expected, it is highest for ESI (at 96.9%), followed by the subsidized marketplace at 92.3% and then the direct/unsubsidized marketplace at 90.2%. Sensitivity drops somewhat when separating into more specific categories. Among the direct/unsubsidized marketplace group, 82.1% are correctly categorized, while 8.1% are incorrectly categorized as having subsidized marketplace plans. Among the subsidized marketplace group, sensitivity drops from 92.3% to 70.5%, with 21.9% being incorrectly classified as having direct/unsubsidized marketplace plans.

On the public side, sensitivity for Medicaid is 83.0% and for MinnesotaCare it is 54.3%. The latter finding is largely owing to the fact that almost 48% of MinnesotaCare enrollees are reported to have direct‐purchase coverage, and another almost 11% are reported to have ESI coverage. Within the Medicaid strata, sensitivity drops from 83.0% (reported to have public coverage) to 69.2% reported to have Medicaid specifically (versus MinnesotaCare). Finally, the MinnesotaCare sensitivity drops from 54.3% with public coverage in general to 37.1% with MinnesotaCare specifically.

We now turn to the analysis of prevalence indicators. Because the data are weighted to represent the known population of enrollees within strata, we can produce a straightforward measure of the prevalence of Coverage Type X as indicated in the enrollment records and compare that to self‐reports in the ACS. As the last column of Table 4 shows, consistent with other literature, private coverage is over‐reported, and public coverage is under‐reported (10.1 and −1.8 percentage points). Within private coverage, direct‐purchase over‐reporting is higher than ESI (7.8 versus 4.8 percentage points), and the over‐reporting of direct/unsubsidized marketplace is driving that difference more so than the subsidized marketplace category. Within public coverage, Medicaid is under‐reported and MinnesotaCare is over‐reported (−4.6 versus 2.7 percentage points).

**TABLE 4:** Prevalence of coverage: Records versus self‐reports (Weighted).

	Enrollment records	ACS self‐reports	Difference (ACS minus records)
Private	**71.3**	**81.4**	**10.1**
ESI	68.1	72.9	4.8
Direct	3.2	10.9	7.8
Direct/Mkt unsubsidized	*3.0*	*8.9*	*5.9*
Mkt subsidized	*0.2*	*2.1*	*1.8*
Public	**23.6**	**21.8**	**−1.8**
Medicaid	*20.2*	15.6	*−4.6*
MinnesotaCare	*3.5*	*6.2*	*2.7*

*Note:* Bold figures are aggregated coverage types (private vs. public). Figures in italics are categories of coverage created using the premium and subsidy items. Due to disclosure avoidance, we do not provide specific sample sizes by strata; see Fertig et al. [11] for more details. Example interpretation: Based on the enrollment records, 71.3% of the sample had private coverage. Based on responses to the ACS survey, 81.4% of the sample had private coverage. Thus, private coverage was overreported by 10.1 percentage points.

Abbreviations: ACS, American Community Survey; ESI, Employer Sponsored Insurance; Mkt, marketplace.

## 4 Discussion and Conclusions

There are several limitations to this study. As mentioned above, the PS sample is slightly biased toward self‐reports and reports for children, thus results may be somewhat biased toward higher reporting accuracy. Second, the study was conducted in only one state, which limits its generalizability. Compared to the rest of the US, Minnesota's population is disproportionately non‐Hispanic White, slightly more educated, and has a higher employment rate and lower poverty rate, all of which are characteristics associated with lower reporting accuracy of health insurance coverage [16]. Third, the CHIME study was conducted via telephone by Census staff, while the majority of production ACS interviews are conducted via internet and mail, and less than 5% are conducted by telephone [17], thus mode effects could be at play. Fourth, because of the small sample that received the experimental premium and subsidy questions, we were not able to conduct sub‐group analyses of reporting accuracy. Fifth, the wording of the premium and subsidy items that was ultimately adopted in production ACS is similar but not verbatim to the wording tested in CHIME. The premium question in CHIME was “Do you or another family member pay a premium for this health insurance plan?” while in the ACS the question is “Is there a premium for this plan?”. For the subsidy item, in CHIME the wording was “Based on family income, do you or another family member receive financial assistance through a subsidy or tax credit to help pay part or all of the cost of the premium for this plan?” and in the ACS the wording is “Does this person or another family member receive a tax credit or subsidy based on family income to help pay the premium?”. Finally, there is the issue of distinguishing sub‐categories of coverage under the umbrella “direct purchase” category. Ideally analysts may prefer three mutually exclusive sub‐categories: conventional non‐group coverage outside the marketplace, marketplace/unsubsidized and marketplace/subsidized coverage. However, production ACS simply does not include the data points needed to separate conventional non‐group from unsubsidized marketplace coverage. While this limits sub‐category categorization, one fortunate shift in the health insurance landscape since the CHIME study was conducted is that the marketplace has eclipsed conventional non‐group coverage outside the marketplace [18]. Thus, the combined conventional non‐group/unsubsidized marketplace category, which represents private, unsubsidized non‐group coverage, is a meaningful category for analysis, particularly when contrasted with the subsidized marketplace and public coverage categories.

The ACS module produces aggregated coverage types (private and public), and within private, ESI and direct purchase. To go beyond that, premium and subsidy items were added to production ACS and we evaluate their accuracy and utility in producing more disaggregated coverage types. Results indicate fairly high levels of reporting accuracy for the two new items on premiums and subsidies. Analysis also indicates that an algorithm leveraging these two new data points can be used to separate the overall “direct purchase” category into two sub‐groups: unsubsidized directly‐purchased coverage (on and off the marketplace) and subsidized marketplace coverage. The new premium item can also be used to separate Medicaid from MinnesotaCare. Reporting accuracy of disaggregated coverage types is generally lower than for more aggregated categories given that aggregated categories mask misreporting of specific coverage types (e.g., respondents could misreport their ESI coverage as non‐group or vice versa, but both would still be accurately categorized as private coverage). Thus, as expected, reporting accuracy drops with the disaggregated categories. For MinnesotaCare the drop is considerable, and it is driven less by misreporting of the premium item and more by misreporting of MinnesotaCare as a private plan (i.e., ESI and/or directly‐purchased), versus a public plan. Overall, these findings should give ACS data users confidence in the accuracy of these new data points and their utility to create more detailed coverage types than previously available.

It is difficult to speculate how any future changes to the Medicaid program, marketplace subsidies, and the messaging around these programs could affect enrollees' understanding of the fundamental source of their coverage (public versus private), premium contribution requirements and subsidies. However, for some cautionary clues we harken back to the results on the subsidy item, where the vast majority of public enrollees reported that the premium was NOT subsidized, but the vast majority of marketplace enrollees correctly reported subsidization. When the ACA and marketplace were rolled out in 2014, there was a heavy emphasis on subsidized premiums. By contrast, in Minnesota, both Medicaid and MinnesotaCare are described as health coverage for people with low incomes and we could find no mention of the term “subsidy” on websites and outreach materials. Perhaps it is no surprise then, that the term “subsidy” is simply not salient to public enrollees, and that lack of saliency is reflected in their survey responses. As of this writing, Medicaid and other public programs use language revolving around concepts such as income eligibility, cost‐sharing and categorical eligibility (e.g., children; young mothers). That kind of language could shift with any fundamental changes to the program (e.g., work requirements). The implications are that survey data analysts should carefully consider the extent to which the marketing and messaging of public programs and marketplace subsidies are reflected in actual survey questions.

## Conflicts of Interest

The authors declare no conflicts of interest.

## Supporting information


**Appendix S1:** Supporting Information.

## Data Availability

The data that support the findings of this study are available from US Census Bureau. Restrictions apply to the availability of these data, which were used under license for this study. Data are available from the author(s) with the permission of US Census Bureau.
