# Supplementary material for: Adapting the American Community Survey for the Affordable Care Act
Source: Health Serv Res. 2025 Nov 4;61(1):e70066. doi: 10.1111/1475-6773.70066 (PMC12857494; doi:10.1111/1475-6773.70066)
Supplement: Supplementary file 1 — Appendix S1: Supporting Information. [file HESR-61-e70066-s001.docx]

**Appendix A: Profile of Sample That Did vs. Did Not Receive Premium/Subsidy (PS) Items**

|  | **Non-PS Sample** | **PS Sample** | **P-value** |
| --- | --- | --- | --- |
| % of ACS sample | 64% | 36% |  |
| **Covered Individual** |  |  |  |
| Female | 56% | 48% | 0.091 |
| Age < 19 | 31% | 42% | 0.081 |
| Age 19-34 | 23% | 17% |  |
| Age 35+ | 45% | 41% |  |
| Self | 48% | 33% | **0.009** |
| Child | 35% | 46% |  |
| Other | 18% | 21% |  |
| Non-Hispanic White | 76% | 79% | 0.618 |
| Non-Hispanic Black | 9% | 7% |  |
| Non-Hispanic Other | 8% | 9% |  |
| Hispanic | 7% | 5% |  |
| Policyholder | 46% | 51% | **0.008** |
| Shared coverage with respondent |  |  |  |
| Missing respondent | 7% | 13% | **<0.001** |
| Proxy report in multi-person household with different coverage | 6% | 8% |  |
| Proxy report in multi-person household with same coverage | 38% | 44% |  |
| Self-report in multi-person household | 38% | 0.05% |  |
| Self-report in one person household | 10% | 35% |  |
| Recency/intensity of coverage |  |  |  |
| Covered at interview and up to 6 months prior | 5% | 8% | 0.122 |
| Covered at interview and 7-17 months prior | 14% | 20% |  |
| Covered at interview and all prior months | 81% | 73% |  |
| **Respondent** |  |  |  |
| < High School | 12% | 10% | 0.366 |
| High School | 21% | 26% |  |
| Some College | 27% | 33% |  |
| 4+ Years of College | 40% | 31% |  |
| Married | 58% | 26% | **<0.001** |
| Widowed/Divorced/Separated | 9% | 13% |  |
| Never Married | 33% | 61% |  |
| Full-time/Full-year employed | 50% | 51% | 0.724 |
| Less than Full-time/Full-year | 37% | 34% |  |
| Not Working | 13% | 15% |  |
| **Household** |  |  |  |
| <200% of Federal Poverty Level | 28% | 21% | **0.021** |
| Single person | 10% | 35% | **<0.001** |
| 2-4 people | 67% | 60% |  |
| 5+ people | 23% | 5% |  |

Note: PS=Sample that received the premium and subsidy items. ACS=American Community Survey.

As noted above, the PS sample includes significantly more children (46% vs. 35%, p=0.009) and significantly more single-person households (35% vs. 10%, p<0.001) than the non-PS sample. We also observe differences in self-reporting vs. proxy-reporting, marital status of the respondent, and policyholder status of the respondent. Specifically, because the PS sample includes more single-person households, there are almost no self-reports in multi-person households (0.05% vs. 38%, p<0.001) in the PS sample, and significantly more self-reports in single-person households (35% vs. 10%, p<0.001) in the PS sample. Similarly, there are also fewer married households in the PS sample (26% vs. 58%, p<0.001) and more policyholders in the PS sample (51% vs. 46%, p=0.008). Finally, the only observed difference that does not obviously follow from the study design is that the PS sample is significantly less likely to have a household income that is <200% of the Federal Poverty Level (FPL) (21% vs. 28%, p=0.021).

**Appendix B: Handling of Write-Ins for the Other/Specify Item**

The ACS allows respondents to select one or more coverage types, and those who select “other” (Question 8 above) are asked to provide more detail in an open-text format. Production ACS maintains a system to back-code these open text responses into substantive coverage type categories^1^. As the authors note, and as we observed, in many cases respondents check the “other” box in addition to a substantive coverage type (e.g., ESI, direct purchase or Medicaid), and then write in the name of a private insurance carrier. This creates ambiguity as it is not clear whether the write-in is meant to simply provide more detail about the coverage reported in Questions 1-7, or whether it represents a plan in addition to one already reported. Regardless of which coverage types(s) is/are selected in Questions 1-7, production ACS generally assigns the private insurance carrier write-ins to the direct purchase category, which can artificially inflate the estimates of non-group coverage.^2^ We examined the open-text write-ins in the CHIME data to explore whether and how to adapt the production ACS back-coding system. Among the small number of write-ins in the CHIME study, very few were ambiguous cases that could have inflated the non-group category. Given this, we mimicked the ACS production method as closely as possible to back-code the other/specify open text responses.

1. Berchick ER, Wiedemann MS. *Health Insurance Coverage and Write-Ins in the American Community Survey*.; 2018. Accessed June 17, 2025. https://www.census.gov/content/dam/Census/library/working-papers/2018/demo/sehsd-wp-2018-03.pdf

2. Mach A, O’Hara B. *Do People Really Have Multiple Health Insurance Plans? Estimates of Nongroup Health Insurance in the American Community Survey*.; 2011. Accessed August 15, 2023. https://www.census.gov/content/dam/Census/library/working-papers/2011/demo/SEHSD-WP2011-28.pdf
